# Latent class trajectories of biochemical parameters and their relationship with risk of mortality in ICU among acute organophosphorus poisoning patients

**DOI:** 10.1038/s41598-022-15973-2

**Published:** 2022-07-08

**Authors:** Waqas Ahmed Farooqui, Mudassir Uddin, Rashid Qadeer, Kashif Shafique

**Affiliations:** 1 School of Public Health, Dow University of Health Sciences, 2nd Floor, Nursing Building, Ojha Campus, Gulzar-e-Hijri, Zohra Nagar, Scheme 33, Karachi City, Sindh Pakistan; 2 Department of Statistics, University of Karachi, Karachi, Pakistan; 3 Department of Medicine, Ruth Pfau Civil Hospital Karachi, Dow University of Health Sciences, Karachi, Pakistan; 4 Institute of Health and Wellbeing, University of Glasgow, Glasgow, UK

**Keywords:** Health care, Medical research, Risk factors

## Abstract

Acute poisoning is a global public health challenge. Several factors played role in high mortality among acute organophosphorus poisoning (OP) poisoning patients including clinical, vitals, and biochemical properties. The traditional analysis techniques use baseline measurements whereas latent profile analysis is a person-centered approach using repeated measurements. To determine varying biochemical parameters and their relationship with intensive care unit (ICU) mortality among acute organophosphorus poisoning patients through a latent class trajectory analysis. The study design was a retrospective cohort and we enrolled data of 299 patients admitted between Aug’10 to Sep’16 to ICU of Dr. Ruth K. M. Pfau, Civil Hospital, Karachi. The dependent variable was ICU-mortality among OP poisoning patients accounting for ICU stay, elapsed time since poison ingestion, age, gender, and biochemical parameters (including electrolytes (potassium, chloride, sodium), creatinine, urea, and random blood sugar). Longitudinal latent profile analysis is used to form the trajectories of parameters. In determining and comparing the risk of ICU-mortality we used Cox-Proportional-Hazards models, repeated measures and trajectories were used as independent variables. The patients’ mean age was 25.4 ± 9.7 years and ICU-mortality was (13.7%, n = 41). In trajectory analysis, patients with trajectories (normal-increasing and high-declining creatinine, high-remitting sodium, normal-increasing, and high-remitting urea) observed the highest ICU-mortality i.e. 75% (6/8), 67% (2/3), 80% (4/5), 75% (6/8), and 67% (2/3) respectively compared with other trajectories. On multivariable analysis, compared with patients who had normal consistent creatinine levels, patients in normal-increasing creatinine class were 15 times [HR:15.2, 95% CI 4.2–54.6], and the high-declining class was 16-times [HR 15.7, 95% CI 3.4–71.6], more likely to die. Patients in with high-remitting sodium, the trajectory was six-times [HR 5.6, 95% CI 2.0–15.8], normal-increasing urea trajectory was four times [HR 3.9, 95% CI 1.4–11.5], and in extremely high-remitting urea trajectory was 15-times [HR 15.4, 95% CI 3.4–69.7], more likely to die compared with those who were in normal-consistent trajectories of sodium and urea respectively. Among OP poisoning patients an increased risk of ICU-mortality were significantly associated with biochemical parameters (sodium, urea, creatinine levels) using latent profile technique.

## Introduction

Acute poisoning is a global public health challenge^1^. The proportion of suicides due to pesticide self-poisoning varies in low- and middle-income countries between regions, from 0.9% in the European region to 48.3% in the Western Pacific region^2^. Among other poisonings, the prevalence of OP poisoning vary in neighboring countries (7.7% in Iran^3^, 20.7% in China and India^4,5^). Among all poisoning cases, the prevalence of OP poisoning was stated closely 46.1%, and due to OP poisoning, 2.7% mortality reported in a study from our city^6^.

Several factors played role in high mortality among acute OP poisoning patients including the age, gender, type, and amount of poison ingested and its biochemical properties, time since ingestion, any pre-existing comorbidities, influence the outcome^7–11^. The prognosis of OP depends on the exposure of toxin, the amount of toxin ingestion, and the physiology of compensation. In our country, it is difficult to judge the amount as patients ingest different brands, which lack the description of concentration of the poisonous substance^6,12^. In clinical settings, the prognosis of these patients is mainly assessed by a variety of different methods including but not limited to vital status, poisoning scoring systems, and laboratory investigations^5,13–22^. Biochemical analysis of blood plays an important role in the diagnosis of intoxicated patients since drugs with biochemical substances produce biochemical changes. Studies have linked biochemical parameters (including amylase, lipase, lactate dehydrogenase (LDH), serum immunoglobulins (IgG, IgA), and creatine phosphokinase (CPK) level) with the severity of OP poisoning but the estimation of these parameters is expensive and most laboratories cannot perform these tests in developing countries^23,24^. Therefore, there is a need to identify simple and widely useable biochemical parameters in assessing the severity of poisoning as well as the prognosis of OP poisoning patients^20^.

Low pseudocholinesterase (PChE), high creatinine (Cr), high sodium (NA^+^), high blood urea nitrogen (BUN), low Glasgow Coma Scale (GCS) scores, and long hospitalization durations have been assessed for their role in OP poisoning patients’ prognosis, but the findings remain inconclusive among OP poisoning patients^25,26^. One of the main reasons might be the conventional approach of using a single baseline measurement of biochemical parameters to predict the mortality of OP poisoning patients. As these biochemical parameters such as level of, random blood sugar, creatinine, blood urea nitrogen, electrolytes, anticholinesterases, red cell distribution width, lactate dehydrogenase, amylase, creatinine kinase, hematocrit, c-reactive protein are dynamic and tend to change substantially over time and also quite dependent on the physiological response of the patient, which vary significantly from patient to patient.

In such a situation, it might not be a suitable method during the follow-up of linking mortality with single measurements at the time of presentation. Because in those studies at baseline only biochemical inquiries were detected, the significant question is, if parameter mean level changes over time, whether that variation leads to some latent classes that are different than the classes made based on a single baseline measurement of the same variable. It might be disposed to to misclassification bias if this single observation method used^1^.

Two of the studies reported biochemical investigations among acute OP poisoning patients used repeated measures, one of the studies discussed the use of home perfusion technique and compare blood glucose level and cholinesterase before and after treatment^27^, while the other study investigated the predictive value of serum acetylcholinesterase levels measured at five different days and its relationship with different neurological syndromes levels^28^.

The classical approach to deal with longitudinal repeated-measures (RM) is analysis of variance (RMANOVA) but new forms of Structural Equation Modeling (SEM) provides new approaches for repeated measure designs^29^.

In such a situation, latent growth modeling (LGM) provides a better alternative to observe and estimate growth trajectories overtime for dynamic variables. SEM advances basic longitudinal analysis of data to include latent variable growth over time while modeling both individual and group changes using slopes and intercepts. When these variables are continuous the technique called longitudinal latent class analysis (LLCA) or more specifically longitudinal latent profile analysis (LLPA)^30^. The traditional analysis techniques are analysis of variance, multiple regression, and multilevel models which are variable-centered approaches whereas LCGA is a person-centered approach focused on identifying unobserved subpopulations comprising similar individuals^31^. To the best of our knowledge, there is no previous study that has compared the repeated measures and latent trajectories of biochemical parameters in OP poisoning patients and their relationship with ICU-mortality.

Therefore, the present study aimed to analyze the growth trajectory of biochemical parameters among OP poisoning patients and comparing two approaches of biochemical parameters individual response (patterns i.e. LLPA vs RM) and their relationship with ICU-mortality using survival analysis.

## Methods

### Data source

All OP poisoning patients admitted during August 2010 to September 2016 at medical ICU of a tertiary care hospital from Karachi-Pakistan i.e. Dr. Ruth K.M. Pfau/Civil Hospital were included in this study. This hospital is one of the largest tertiary care hospitals in the province of Sindh—Pakistan with an annual patient turnover of approximately > 4,000,000.

### Study participants

A total of 299 OP poisoning patients’ older than 13 years of age and whose biochemical parameter data were available were included in this study. Only those patients were included in this study whose at least two to four observations of biochemical parameters with 12–24 h intervals within 0–96 h of admission were available in medical records. The initial day of follow-up was the beginning of the fifth day in ICU (since they used the first 4-day data to construct latent class). Every study subject had posterior probabilities for each latent class, we calculated the odd of the highest to the lowest posterior probability and report the proportion in study samples with an odd larger than 5.

### Study design and sample

This was a retrospective cohort study on OP poisoning patients who was shifted to ICU.

### Data collection tool

A proforma was used to retrieve demographic, clinical characteristics, and biochemical parameters’ information.

### Ethical consideration

The ethical approval has been granted by the Institutional Review Board of Dow University of Health Sciences (DUHS) (Ref No. IRB-560/DUHS/Approval/2015/75 dated 11th Jun 2015) with an exemption from requiring written informed consent. All methods were carried out in accordance with relevant guidelines and regulations.

### Study variables

Each patient’s data was obtained from medical records.

#### Independent variables

Demographics (age, gender), elapse time since poison ingestion and ICU stay. In addition to these variables, biochemical parameters including random blood sugar, creatinine, blood urea nitrogen, sodium electrolyte, chloride electrolyte, potassium electrolyte were also retrieved for an initial period of 4 days as per completion of medical records. These biochemical parameters were captured overtime during the hospital stay.

#### Dependent variable

ICU-mortality data were recorded to assess the outcome of patients within ICU stay.

### Statistical analysis

The data were entered and managed using MICROSOFT OFFICE 365 EXCEL. LLPA was utilized for identifying unknown latent classes of individuals with respect to measurements at different time points. It does not model changes over time, but the patterns of measures that span multiple time points^31^. LLPA used in MPLUS version 7.0 software to detect patterns of biochemical parameters. Models were prepared as per guidelines^31,32^. Our models used first-order polynomials observed linear growth pattern and for each class (two/three/four) whichever best on fit indices to find the maximum latent classes. For every individual, we computed the posterior probabilities and latent class. We choose classes based on each latent class proportion size of at least 5 cases (1.5%) and best-fitting high values of Log-likelihood, Lo–Mendell–Rubin Test, Bootstrap Likelihood-Ratio Test, low values of Akaike Information Criterion (AIC), Bayesian Information Criterion (BIC), sample size adjusted BIC^33^.

**Figure 1:**
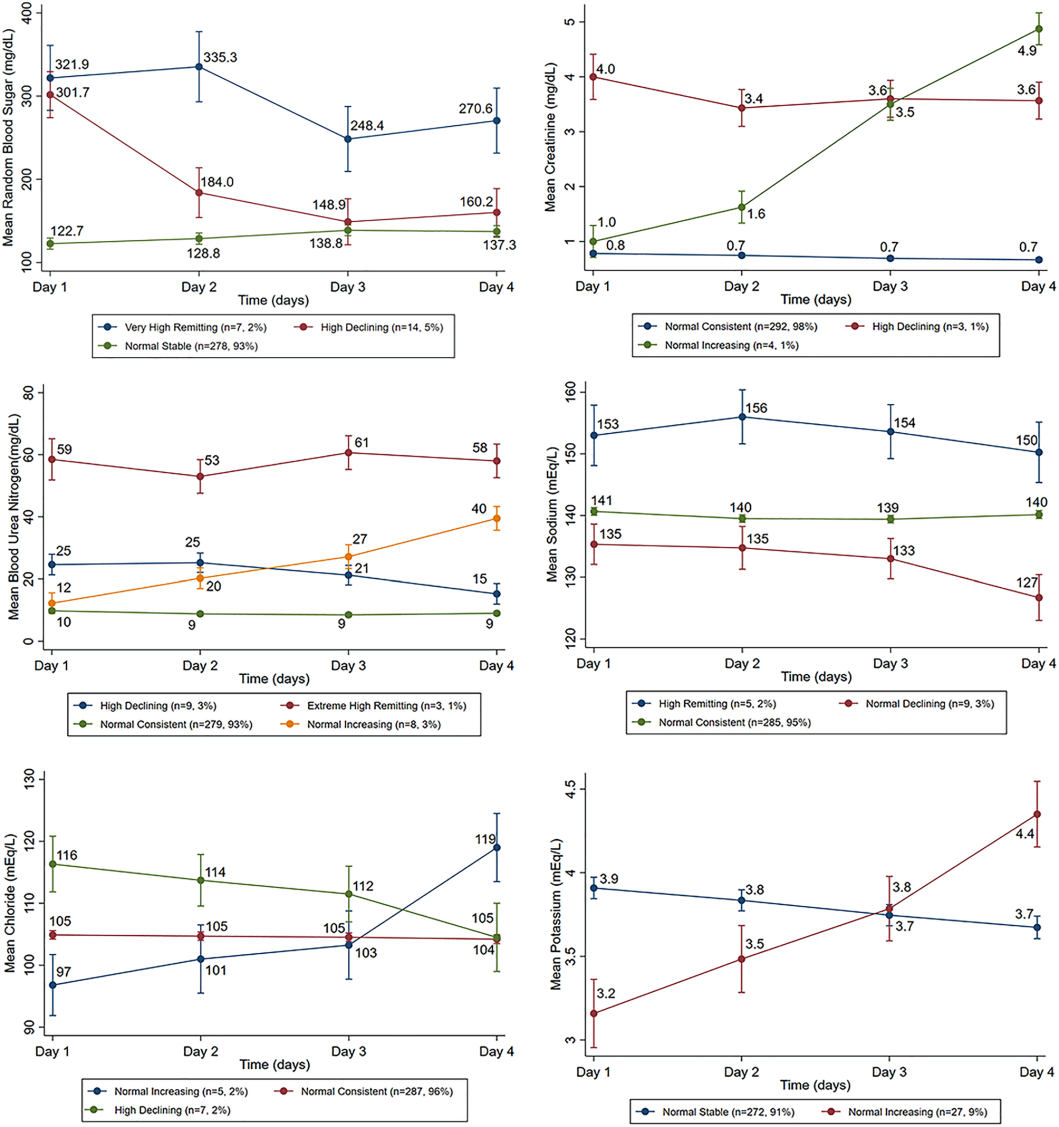
Trajectories of biochemical parameters from day 1 to 4.

Based on the modeled graphical shapes, labels were assigned to the trajectories (Fig. 1). These were based on slope and intercept of mean of individual’s pattern. Based on the start of biochemical parameters ranges, we defined labels first part (e.g. low, normal, high, extreme) and on their patterns of repeated measure we identified second part (e.g. consistent, stable, declining, increasing, remitting (up then down or vice versa)). Form of multiple repetition of high and low observations refers to the term remitting during the course of observations i.e. 4 days starting from high to low or vice versa^1^.

For inferential analysis and figures used STATA version 15.0 software (Stata Corp, College Station, Texas, USA). To detect differences in age, total ICU stays and baseline biochemical parameters between dead and alive patients, Wilcoxon rank-sum test was applied. Association between gender and biochemical parameters latent classes with ICU-mortality were assessed using chi-square and Fisher exact test where appropriate.

For survival analysis, total ICU stay time was used to define risk time. We computed unadjusted and adjusted hazard ratios (HR) and respective confidence intervals for ICU-mortality and at the same time compared two approaches using independent variables as assigned trajectory and, in another approach taking independent variables as repeated measures. Cox proportional hazards model was applied. In multivariable analysis, we adjusted for age and approximate time elapsed since ingestion of poison. We evaluated the proportional hazards assumption of Schoenfeld residuals by phtest and power of the Cox Model using Harrell's C concordance statistic; assumptions were identified satisfactory and not violated^34,35^. P-values < 0.05 were considered statistically significant.

### Ethics approval and consent to participate

The ethical approval has been granted by the Institutional Review Board of Dow University of Health Sciences (DUHS) (Ref No. IRB-560/DUHS/Approval/2015/75 dated 11th Jun 2015) since teaching Hospital Civil is affiliated with DUHS with an exemption from requiring written informed consent.

## Results

A total of 499 OP poisoning patients (of either gender) were eligible during the 6 months data collection period from June 2016 to November 2016. Out of the total, 200 patients’ records were excluded due to incomplete data, wrong registration, self-reported history of chronic conditions such as hypertension, diabetes mellitus, osteoarthritis, asthma, or pregnant women, less than 3 days ICU stay. A total of 299 patients’ data was included for final analysis with a mean ± standard deviation age of cohort 25.4 ± 9.7 years (ranged 13–70 years) with an overall ICU-mortality of 13.7% (n = 41). Biochemical parameters (including random blood sugar, creatinine, blood urea nitrogen), age, elapsed time since ingestion of poison were significantly higher among dead as compared to alive individuals, while gender, total ICU stay, and electrolytes were not significantly different between dead and alive individuals (Table 1).

**Table 1:** Descriptive statistics of baseline characteristics with ICU-mortality.

Characteristics	Alive (N = 258, 86.3%)	Dead (N = 41, 13.7%)	P-value
**Gender**			
Female	131 (87.3)	19 (12.7)	0.598^~^
Male	127 (85.1)	22 (14.9)	
Age (years)	23 (18–28)	27 (20–40)	0.009^†^
Elapse time (hours)	5.2 (2.5–8.4)	10 (8.0–12.5)	< 0.001^†^
ICU stay (days)	5.7 (3.8–10.8)	7.5 (4.1–10.5)	0.519^†^
**Biochemical**			
RBS (mg/dL)	113.0 (100.0–151.0)	143.0 (105.0–185.0)	0.018^†^
Cr (mg/dL)	0.7 (0.6–0.9)	0.9 (0.7–1.1)	0.009^†^
BUN (mg/dL)	10 (7–12)	11 (9–15)	0.012^†^
**Electrolytes**			
Sodium (Na^+^) (mEq/L)	141 (138–143)	140 (137–143)	0.600^†^
Chloride (Cl^-^) (mEq/L)	105 (102–108)	104 (101–107.5)	0.555^†^
Potassium (K^+^) (mEq/L)	3.9 (3.5–4.1)	3.9 (3.5–4.1)	0.564^†^

*IQR* interquartile range (25th–75th percentile); ^†^Wilcoxon Rank Sum Test;^~^Chi-square Test, *ICU* intensive care unit, *RBS* random blood sugar, *Cr* creatinine, *BUN* blood urea nitrogen.

Three distinct trajectories of creatinine (normal-consistent, high-declining and normal-increasing) were identified among 299 OP poisoning patients (Fig. 1), ICU-mortality in normal-consistent trajectory was 36/292 = 12.3%, in normal-increasing was 03/04 = 75.0%, while ICU-mortality in high-declining trajectory was 02/03 = 66.7%) (Table 2).

**Table 2:** ICU-mortality comparison of biochemical parameters—latent classes.

Parameters	Total (N = 299)	Alive N = 258 (%)	Dead N = 41 (%)	P-value^^^
**RBS**				
Normal stable	278	243 (87.4)	35 (12.6)	0.070
Very high remitting	07	05 (71.4)	02 (28.6)	
High declining	14	10 (71.4)	04 (28.6)	
**Creatinine**				
Normal consistent	292	256 (87.7)	36 (12.3)	0.001
High declining	03	01 (33.3)	02 (66.7)	
Normal increasing	04	01 (25.0)	03 (75.0)	
**Blood urea nitrogen**				
Normal consistent	279	250 (89.6)	29 (10.4)	< 0.001
High declining	09	05 (55.6)	04 (44.4)	
Extremely high remitting	03	01 (33.3)	02 (66.7)	
Normal increasing	08	02 (25.0)	06 (75.0)	
**Sodium**				
Normal consistent	285	248 (87.0)	37 (13.0)	0.002
High remitting	05	01 (20.0)	04 (80.0)	
Normal declining	09	9 (100.0)	0 (0)	
**Chloride**				
Normal consistent	287	250 (87.1)	37 (12.9)	0.073
Normal increasing	05	03 (60.0)	02 (40.0)	
High declining	07	05 (71.4)	02 (28.6)	
**Potassium**				
Normal stable	272	236 (86.8)	36 (13.2)	0.392
Normal increasing	27	22 (81.5)	05 (18.5)	

^^^Fisher exact test.

Four trajectories of urea were identified (normal-consistent, high-declining, extreme high remitting, and normal-increasing) (Fig. 1). Patients’ ICU-mortality in normal-consistent trajectory was 29/279 = 10.4%, in high-declining was 04/09 = 44.4%, in extremely high-remitting was 02/03 = 66.7%, while ICU-mortality in normal-increasing trajectory was 06/08 = 75.0%) (Table 2).

Three trajectories were identified for sodium electrolyte (normal-consistent, high-remitting, and normal declining) (Fig. 1). Patients’ ICU-mortality in normal-consistent trajectory was 37/285 = 13.0%, in extremely high-remitting it was 04/05 = 80.0%, while ICU-mortality in normal declining trajectory was 0/9 = 0% (Table 2).

On multivariable analysis after adjusting for age, when modeled repeated measures, creatinine increased the risk of ICU-mortality [HR 1.17, 95% CI 1.12–1.22, P < 0.001]. However, on multivariable analysis after adjusting for age and elapsed time when modeled latent classes, patients with high-declining creatinine were 16 times more likely [HR 15.7, 95% CI 3.4–71.6, P < 0.001] and normal-increasing were 15 times more likely [HR 15.2, 95% CI 4.2–54.6, P < 0.001] to die compared with those who were in normal-consistent trajectory. In the multivariable model, repeated measures analysis, urea showed a significant relation with ICU-mortality [HR 1.01, 95% CI 1.01–1.02, P < 0.001]. However, while using latent classes, patients in high-remitting urea trajectory were 15 times [HR 15.4, 95% CI 3.4–69.7, P < 0.001] and in normal-increasing urea trajectory were four times [HR 3.9, 95% CI 1.4–11.5, P = 0.012] more likely to die compared with those who had normal-consistent urea. In multivariable model repeated measures analysis, sodium did not show increased risk of ICU-mortality [HR 1.0, 95% CI 0.999–1.001, P = 0.958]. However, on latent classes, patients in high-remitting sodium trajectory were six times [HR 5.6, 95% CI 2.0–15.8, P = 0.001] more likely to die compared with those who had normal consistent sodium (Table 3).

**Table 3:** Two approaches in relationship of ICU-mortality with biochemical parameters using Cox model.

Parameters	Unadjusted HR (95% CI)	Adjusted HR (95% CI)
**Repeated measures approach**		
Random blood sugar (mg/dL)	1.0 (0.999, 1.001)	1.0 (0.999, 1.001)
Creatinine (mg/dL)	1.18 (1.13, 1.22)	1.17 (1.12, 1.22)
Blood urea nitrogen (mg/dL)	1.02 (1.01, 1.02)	1.01 (1.01, 1.02)
Electrolytes—sodium(mEq/L)	1.0 (0.997, 1.002)	1.0 (0.999, 1.001)
Electrolytes—chloride(mEq/L)	1.0 (0.996, 1.003)	1.0 (0.997, 1.001)
Electrolytes—potassium (mEq/L)	1.01 (0.93, 1.11)	1.01 (0.98, 1.05)
**Latent class approach**		
RBS		
Normal stable	1.0	1.0
Very high remitting	2.1 (0.5, 8.6)	2.9 (0.7, 12.3)
High declining	1.9 (0.7, 5.4)	1.0 (0.3, 3.2)
Creatinine		
Normal consistent	1.0	1.0
High declining	11.6 (2.7, 49.6)	15.7 (3.4, 71.6)
Normal increasing	17.5 (5.1, 60.5)	15.2 (4.2, 54.6)
Blood urea nitrogen		
Normal consistent	1.0	1.0
High declining	3.5 (1.2, 10)	2.4 (0.8, 7.0)
Extremely high remitting	13.1 (3.0, 55.9)	15.4 (3.4, 69.7)
Normal increasing	8.2 (3.3, 20.4)	3.9 (1.4, 11.5)
Electrolyte—sodium		
Normal consistent	1.0	1.0
High remitting	6.2 (2.2, 17.4)	5.6 (2.0, 15.8)
Normal declining	–	–
Electrolyte—chloride		
Normal consistent	1.0	1.0
Normal increasing	2.0 (0.4, 11.0)	2.4 (0.4, 12.8)
High declining	2.3 (0.5, 9.5)	2.7 (0.6, 11.9)
Electrolyte—potassium		
Normal stable	1.0	1.0
Normal increasing	0.9 (0.3, 2.3)	0.88 (0.46, 1.68)

*HR* hazard ratio.

Adjusted covariates: (for repeated measure: age, for latent class: age and elapse time).

## Discussion

### Summary of findings

Among OP poisoning cases, higher age and longer elapsed time since ingestion were significantly associated with ICU-mortality. Patients in high-declining and in normal-increasing creatinine trajectories and those who were in extremely high-remitting and normal-increasing blood urea trajectories, had significantly high ICU-mortality compared to patients in normal consistent creatinine trajectory and urea trajectory respectively. Additionally, high-remitting sodium trajectory has significant ICU-mortality compared to individuals in normal consistent sodium level trajectory.

The ICU-mortality in our study is comparable to previously published papers on OP poisoning from different regions including neighbor countries^5,6,13,26,36–38^, however our study appeared to show higher (13.7%) ICU-mortality compared to another recently published paper (2.7%) from National Poisoning Control Centre of our urban city. Low mortality in the previous study was perhaps because the study had only 6 months records available [46.1% (1174/2546)] for those OP poisoning patients who were managed in medical wards^6^ whereas our study spanned over 6 years patients data recorded from OP poisoning patients admitted in ICU. Longer time elapsed since ingestion of poison and higher age was significant predictors of mortality and findings are quite consistent with previously published studies^13,26,36^.

Trajectory analysis showed that two trajectories of creatinine (including high-declining and normal increasing) had a significant relationship with ICU-mortality among OP poisoning patients. These results were consistent with previous studies as high creatinine found a significant factor of mortality in multiple Asian studies from 2013 to 2018 including one of our urban cities^13,17,22,26,39^. Two of Asian studies reported risk of mortality on univariable analysis, patients having high creatinine level founds seven to eight times more likely to die^21,40^, whereas in our study patients in normal-increasing trajectory were 18 times and high-declining were twelve times more likely to die compared to the normal consistent creatinine level. This may be due to the mortality within 30 days in these studies whereas in our study it was 20 days.

Trajectory analysis also shown that two trajectories of blood urea nitrogen (including extremely high-remitting and normal increasing) were significantly associated with ICU-mortality among OP poisoning patients. Our study showed four times and 15 times increased risk of ICU-mortality for normal increasing and extremely high remitting trajectories patients respectively, when compared with normal consistent urea trajectory. These results were somewhat supported by two Asian studies which shown high urea level was associated with mortality^17,39^, and were contrasting with few European and Asian studies which shown urea was not a significant factor of mortality^13,22,26^. The LLPA shown in our study that high-remitting trajectory of sodium electrolyte was significantly associated with increased ICU-mortality among OP poisoning patients, however previous evidence shown no relationship between sodium and mortality among OP poisoning patients^13,26^. The difference of urea and sodium may be due to data collection from the emergency department and a single baseline reading in these studies. The results of random blood sugar from our analysis and previous evidence remains consistent showing no relationship with mortality^13,26,27^.

In a comparison of the two techniques repeated measures and latent trajectories, on multivariable analysis, when modeled repeated measure creatinine, and urea level showed a significant relationship with low risk of ICU-mortality, while sodium level does not increase the risk of ICU-mortality. However, when modeled latent classes patients in high-declining creatinine class were 16 times, normal-increasing was 15 times more likely to die compared with those who had normal-consistent creatinine level. Patients in extremely high-remitting urea trajectory was 15 times, normal-increasing urea trajectory were four times and in high-remitting sodium trajectory were six times more likely to die compared with those who were in normal-consistent trajectories of urea and sodium respectively. This reveals that repeated measure approach is predicted low risk of ICU-mortality using creatinine and urea levels while latent class trajectories predicted a high risk of ICU-mortality using creatinine, urea, and sodium levels.

Previous studies only included baseline measurements of biochemical parameters and examined their relationship with mortality, while this study was unique in the sense that it accounted for multiple observations of biochemical parameters in the first 4 days of poisoning and linked them with ICU-mortality. Our approach is also closer to real-life scenario, where such OP poisoning patients have varying levels of these parameters, therefore the findings can have more application in clinical settings. This study has examined more accessible, relatively inexpensive biochemical parameters and shown more clinical utility and are much more convenient than expensive laboratory-based markers and may be considered for use in clinical settings for future OP poisoning patients admitted for intensive care. Commonly used scoring systems heavily depends on clinical and laboratory investigational information^5,17,18,41–45^, and rely on single measurements usually at the time of admission of patient. LLPA used in this study is a person-centered approach where latent classes identify trajectories of patients based on repeated measures of very routine clinical parameters^29^.

### Strengths and weakness

This study included multiple observations of biochemical parameters in the first 4 days of patients’ admission into ICU and determined the relationship of different trajectories of biochemical parameters with ICU-mortality among OP poisoning patients. To the best of our knowledge, this is the first study that determined the relationship of biochemical parameters with ICU-mortality among OP poisoning patients using latent profile approach. To the best of our knowledge, this is the first study that compared the two statistical approaches i.e. latent profile research and repeated measures analysis to determine the risk of ICU-mortality among OP poisoning patients. The current study had a reasonably larger sample size by including data of several years from ICU of a tertiary care hospital, which provided generalizable results in our context.

The current study had an estimated amount of poison ingested from medical records, which might not have been very accurate in terms of the actual amount ingested by the patient. Our study was only limited to routinely performed biochemical analysis among OP poisoning patients and could not include arterial blood gas such as base deficit and other biomarkers such as anticholinesterases, lactate dehydrogenase because the repeated measures of these markers were not available in medical records for trajectory analysis. This study may have survival selection bias because patients who did not survive beyond the first 4 days in ICU were not included in the analysis. The clinical application of the latent profile technique may be limited given the relatively short ICU stay and the requirement of 4 days to identify a distinctive trajectory.

## Conclusion

Our study shows that the latent classes of biochemical parameters, high-declining and normal-increasing trajectories for creatinine, extremely high-remitting and normal-increasing trajectories of blood urea nitrogen, and high-remitting trajectory of sodium electrolyte are significant predictors of ICU-mortality among acute OP poisoning patients. The latent profile technique appeared to provide better results and prediction compared to conventional repeated measure analysis for such OP poisoning patients.

## Data Availability

Data included in the current study are not publicly available to ensure confidentiality of the patients but are available from the corresponding author on reasonable request.

## Abbreviations

OP: Organophosphorus
LLCA: Longitudinal latent class analysis
LLPA: Longitudinal latent profile analysis
LCGA: Latent class growth analysis
BUN: Blood urea nitrogen
PChE: Pseudocholinesterase
Cr: Creatinine
NA^+^: Sodium
RBS: Random blood sugar
IQR: Inter-quartile range
ICU: Intensive care unit
GCS: Glasgow Coma Scale
AIC: Akaike information criteria
BIC: Bayesian information criteria
HR: Hazard ratio
RMANOVA: Repeated-measures analysis of variance
SEM: Structural equation models
